# Public Medical Insurance and Healthcare Utilization and Expenditures of Older with Chronic Diseases in Rural China: Evidence from NRCMS

**DOI:** 10.3390/ijerph17207683

**Published:** 2020-10-21

**Authors:** Qi Liu, Jin Liu, Shuntian Sui

**Affiliations:** 1 School of International Economics and Trade, Shanghai Lixin University of Accounting and Finance, 995 Shangchuan Road, Shanghai 201209, China; qi-liu@lixin.edu.cn; 2 Research Institute for Agriculture, Farmer and Rural Society in China, Shanghai University of Finance and Economics, 777 Guoding Road, Shanghai 200433, China; suishuntian@hotmail.com; 3 School of Urban and Regional Science, Shanghai University of Finance and Economics, 777 Guoding Road, Shanghai 200433, China

**Keywords:** public health insurance, chronic diseases, health-care utilization, health-care expenditures, older

## Abstract

China’s rural older are the threat from chronic diseases, making it important to evaluate the effect of public health insurance on the health care utilization and expenditures with chronic diseases. In 2003, China initiated a public health insurance, which was called the New Rural Cooperative Medical System (NRCMS). NRCMS is a voluntary program, targeting rural residents with government subsidies and individual contribution. Using the two-stage residual inclusion approach (2SRI), we analyzed the impact of NRCMS on health-care service utilization and expenditure of rural older with chronic diseases by using the 2011 and 2013 China Health and Retirement Survey (CHARLS) data. The results showed NRCMS did not play an effective role on improving the medical services utilization of rural older with chronic diseases. Although NRCMS immediate reimbursement significantly reduced the outpatient service fee, the actual outpatient reimbursement is the opposite. In addition, NRCMS did not significantly decrease their hospitalization expense. Policy makers should pay attention to health management about chronic diseases in rural China, and some measures should be taken to deepen the medical security system reform and improve the public health service system.

## 1. Introduction

China has transitioned from being one of the fastest-growing populations to among the most rapidly aging countries worldwide [1]. More than 260 million people suffer from chronic non-communicable diseases, including cardiovascular diseases, cancer, chronic respiratory diseases, psychosis and diabetes mellitus. Based on the data from National Health and Family Planning Commission of the People’s Republic of China: Interpretation of China’s Medium-and Long-term Plan for the Prevention and Treatment of Chronic Diseases (2017–2025), chronic non-communicable diseases have caused 86.6% of the total deaths and more than 70% of the total disease burden in China. It has become the main health threat to people, especially the rural elderly population, and their families. In rural China, elderly people have a high demand for health care or medical services. However, they also have to reduce the utilization of medical services resulting from the high medical costs. This issue continues to pose a huge challenge to health equity and social justice [2]. Meanwhile, due to the underdeveloped public health and the ineffective chronic disease management in rural areas, the elderly and their families are facing a heavy financial burden [3,4]. Because of the higher hospital copayments required under insurance coverage for rural citizens, they were more than twice as likely to drop out of treatment than Chinese in urban areas [5]. The heavy financial burden leads to the life quality deterioration of the elderly in rural area. Some studies have pointed the rural elderly population even commit suicide in order to end their suffering [6,7,8,9,10,11]. Illness is one of the root causes of poverty in rural China [12]. If health intervention is not carried out in time, therefore, it will not only aggravate the burden on their families, increasing the risk of poverty caused by ageing and illness, but also cause other social problems, making it more difficult to alleviate poverty precisely, and hindering the sharing of social development achievements with the rural elderly.

Public health or medical insurance can increase the elderly usage of social health care services in many ways, and influence the demand and control the cost for health care, which can improve the health of the elderly. In 2003, China initiated the New Rural Cooperative Medical System (NRCMS). NRCMS is a voluntary program, targeting rural residents with government subsidies and individual contribution. As Yip and Hsiao, You and Kobayashi pointed, the voluntary feature was adopted to overcome public mistrust in the government-run insurance program [13,14]. As the main public health insurance in rural China, does the New Rural Cooperative Medical Scheme (NRCMS) improve the utilization of medical services for elderly chronically ill patients? Does it reduce the medical care cost for the rural family? These questions need to be answered through in-depth empirical analysis. Obviously, in the context of the high incidence of chronic diseases, it is very important to access the medical care service for rural older, promote healthy aging, evaluate the effect of NRCMS and promote the construction of healthy China.

The literature has examined the effect of NRCMS on medical service utilization, health improvement, medical treatment behavior, medical cost burden and agricultural labor supply. Babiarz et al. found NRCMS was associated with a 26% increase in weekly patient flow, and for individuals, participation in NRCMS was associated with a 5% increase in village clinic use [15]. Zhong found that immediate reimbursement of NRCMS significantly increased the likelihood of patients seeking outpatient treatment in China [16]. Cheng et al. used the data of Chinese Longitudinal Healthy Longevity Survey (CLHLS) in 2005 and 2008 to investigate the performance of NRCMS [4]. Their results showed that NRCMS improved the health status of the elderly and increased the medical service utilization rate. More studies have shown that NRCMS is effective in increasing access to care [17,18], reducing health expenditure [19,20] and improving labor supply [21]. However, there still is skeptical that NRCMS has not reduced their actual medical expenditure [22]. Brown et al. insisted that, conditional on seeking treatment, the reimbursement scheme of NRCMS and average daily costs strongly influenced hospital choice [23]. A study showed the effect of the NCMS reimbursement policies for chronic disease on catastrophic health expenditure was negative [24]. These studies show that the program has also encouraged farmers to get more health care, resulting from moral hazard and adverse selection in health insurance and care utilization. differences in health care utilization and costs between those with and without social health insurance and between those with different health insurance schemes [25]. Hence, there still is a need for local government to continue to take effective countermeasures.

Our paper argues that although there are many empirical studies focus on the effect of NRCMS, there exists some shortages as following. First, when examining the medical service utilization and NRCMS expenditure, we know little about the impact of NRCMS on medical service utilization for rural older with chronic diseases. Secondly, different conclusions have been drawn due to different understanding of the actual situation in China, different data sources and different research methods. The views on whether NRCMS can improve the medical service utilization and reduce medical expenditure of participating farmers still show great differences, disputes, and even contradictions, which does not help to accurately understand the effect of NRCMS policy.

From the perspective of chronic diseases, we empirically analyze the effect of NRCMS on medical service utilization and medical expenditure among rural older by using the data of CHARLS in 2011 and 2013. Compared with previous studies, the main contributions of this paper are as follows. Firstly, we focus on the influence of NRCMS on medical service utilization and medical expenditure for older with chronic diseases in rural China. The conclusion enriches the research on NRCMS. Secondly, regarding the empirical method, the four-part model method is used to overcome the shortcomings of the previous research methods on medical service utilization and expenditure, and the two-stage residual inclusion approach (2SRI) is used to deal with endogenous problems, which ensures the reliability of the conclusions.

The rest parts are arranged as follows: the second part is the empirical model setting and description of data and variables; the third part is the empirical analysis and results discussion; the fourth part is the conclusion and policy implications.

## 2. Empirical Method, Data, and Variables

### 2.1. Empirical Method

Personal medical expenditure is a continuous variable with an interval of [0, +∞). Its distribution has at least three characteristics that make it unsuitable for general regression analysis. First, about 20% of the population has no medical care expenditure in a given year; second, 80% of the population has positive medical expenditures, and medical expenditures are highly biased; third, while 10% of the population has hospitalization expenditures, the right tail of the distribution of medical expenditures does not have a normal distribution [26]. In brief, the highly skewed distribution of medical expenditures, with a large number of non-spenders, makes reliable estimation and prediction difficult [26]. Traditional linear regression methods are not suitable to analyze the residents’ medical service utilization.

Duan et al. [26] reviewed alternative models for cross-sectional medical cost data. Specifically, they discussed three models often used in health economics: (1) a one-part model which utilizes a two-parameter Box–Cox transformation of expenditures; (2) a two-part model with a pair of equations-a probit model for the probability of cost being positive and a linear model for the level of the (log transformed) positive cost; (3) a four-part model to further distinguish inpatient and outpatient costs, with four separate equations. This four-part model describes: (a) the probability of a positive expense; (b) the probability of a positive inpatient expense conditional on a positive expenditure; (c) the level of expense for those with only outpatient medical service; and (d) the level of expense for those hospitalized. They applied these models to a large portion of the Rand Health Insurance Experiment (RHIE) data and found that both the two-part model and the four-part model performed better than the one-part model in terms of consistency and accuracy for making forecasts. They expected the four-part model would excel the two-part model if more data were available, since the two-part model has been shown to lead to inconsistent predictions [27].

In this paper, the four-part model method is adopted to investigate the influence of NRCMS on the medical service utilization and medical expenditure of rural elderly chronic disease patients in China. It divides the research sample into three groups: zero medical expenditure group (with no medical expenditure in a given period of time), outpatient group, and hospitalization group. Logarithmic transformation of non-zero medical expenditure is done to make the distribution of medical expenditure unbiased, consistent and approximate positive, leading to a more accurate estimation [26]. The specifics are as follows:

Model 1: Probability model of medical treatment choice of chronic disease patients, namely ‘whether to seek medical treatment’ model. This model is about the probability model of choosing medical treatment for observed samples in a given time. It distinguishes whether to see a doctor or not, which is the basis of investigating the medical service utilization and the cost burden of the observed samples. In this model, the probability ($hos_{1i}$) of the of occurred medical treatment (including outpatient and hospitalization) in a given time is expressed as follows:

(1) $$hos_{1i}=X_{i}\beta_{1}+\varepsilon_{1i}$$

In Formula (1), $hos_{1i}>0$ means there is medical service utilization and medical expenditure; otherwise it is 0. $\varepsilon_{1i}\sim N\;\left(0,1\right)$, this part takes all the samples and the samples participating in the NRCMS as the research object, and investigates the influencing factors of the decision-making of the elderly chronic disease patients. Because this part is divided into ‘choose to see a doctor’ and ‘not choose to see a doctor’, which are set as a ‘0–1’ variable, the binary probit regression model is adopted.

Model 2: Probability model of hospitalization choice, i.e., for the sample seeking medical treatment, whether they choose outpatient or hospitalization is also a ‘0–1’ variable, and it is expressed as follows:

(2) $$hos_{2i}=X_{i}\beta_{1}+\varepsilon_{1i}$$

In Formula (2), $\varepsilon_{2i}\sim N\;\left(0,1\right)$. Same as model 1, the dependent variable is a binary variable, so the binary probit regression model is used again.

Model 3: Medical expenditure model of outpatient samples is expressed as follows:

(3) $$Med_{1i}{|}_{hos_{1i}>0\;and\;hos_{2i}=0}=X_{i}\beta_{3}+\varepsilon_{3i}$$

In Formula (3), $\varepsilon_{3i}\sim N\;\left(0,\sigma_{\varepsilon_{3i}}^{2}\right)$.

Model 4: Medical expenditure model of hospitalization samples is expressed as follows:

(4) $$Med_{2i}{|}_{hos_{1i}>0\;and\;hos_{2i}=1}=X_{i}\beta_{4}+\varepsilon_{4i}$$

In Formula (4), $\varepsilon_{4i}\sim N\;\left(0,\sigma_{\varepsilon_{4i}}^{2}\right)$.

Because the dependent variables in model 3 and 4 belong to continuous variables, the least square method is used for regression. In Formulas (1)–(4): $hos_{i}$ means individual i’s decision-making on medical behavior, including whether to seek medical treatment, and whether to choose hospitalization or outpatient in the treatment; $Med_{i}$ refers to individual $i^{\prime }$ medical expenditure burden in medical treatment; $X_{i}$ represents variables of social demographic and economic characteristics of NRCMS, number of visits, travel distance, individual characteristics and family characteristics; ε is the error term.

As NRCMS emphasizes the voluntary participation principle, the participation of the rural elderly is not an exogenous random decision, but is influenced by economic conditions, health and other factors with endogenous nature. A common solution is the instrumental variable method. A commonly used instrumental variable selection method is to adopt macro-level variables, such as the provincial NRCMS participation rate published annually by the Ministry of Human Resources and Social Security of China. Calculated by dividing the number of participants by the number of household registrations, it is related to the decision-making on individual participation, but not directly related to the decision-making on individual medical service utilization and expenditure. Therefore, this paper employs the provincial participation rate as an instrumental variable.

In addition, the instrumental variable method can be implemented by 2SRI. Actually, in health economics research, dependent variables are often limited dependent variables, counting variables or skewed distribution, etc. As a consistent and effective estimation method to solve the endogenous problem of non-linear models, 2SRI is widely used in health economics researches [28,29,30,31]. In the meantime, in order to ensure the robustness and consistency of the results, 500 times bootstrapping will be used to estimate the regression parameters.

### 2.2. Data Sources and Main Variables

The primary database used in this work is drawn from the China Health and Retirement Longitudinal Study (CHARLS). CHARLS is a nationally representative longitudinal survey of persons in China 45 years of age or older and their spouses, including assessments of social, economic, and health circumstances of community residents [32]. All data will be made public one year after the end of data collection. CHARLS adopts multi-stage stratified PPS sampling. As an innovation of CHARLS, a software package (CHARLS-GIS, CHARLS, Peking University, Beijing, China) is being created to make village sampling frames. The baseline national wave of CHARLS is being fielded in 2011 and includes about 10,000 households and 17,500 individuals in 150 counties/districts and 450 villages/resident committees (or villages) from 28 provinces. Furthermore, the CHARLS respondents are followed up every two years, using a face-to-face computer-assisted personal interview. More details are offered by Zhao et al. [32].

This study uses the data of CHARLS in 2011 and 2013, which is a survey conducted in 28 provinces in China in 2011 and 2013 with people over 45 years old and their spouses as the subject, getting final samples of 17,708 and 18,605, respectively. Since we are interested in exploring the relationship between the health of the elderly and the social engagement, we restrict our attention to the subsample of the elderly in China, and further limit our sample to respondents who are aged 60 or above. After eliminating the missing key variables and the urban samples classified by the National Bureau of Statistics, 2418 samples are used, of which 1411 and 1007 are from 2011 and 2013, respectively. The main variables involved in this study are as follows:

#### 2.2.1. Chronic Diseases

In the CHARLS questionnaire, the chronic disease information of main respondents can be obtained by asking ‘Did a doctor ever tell you that you had the following chronic diseases, including hypertension, dyslipidemia (elevation of low density lipoprotein, triglycerides and total cholesterol, or a low high density lipoprotein level), diabetes or high blood sugar, cancer or malignant tumor (excluding minor skin cancers), chronic lung diseases (such as chronic bronchitis, emphysema but excluding tumors or cancer, liver disease (except fatty liver, tumors and cancer), heart attack (including coronary heart disease, angina, congestive heart failure, or other heart problems), stroke, kidney disease (except for tumor or cancer); Stomach or other digestive disease (except for tumor or cancer), emotional (nervous or psychiatric) problems, memory-related disease, arthritis or rheumatism, asthma’, ‘Did you know you have hypertension, chronic lung disease, emotional and mental problems?’ If the main respondent is informed by the doctor and knows that he has these chronic diseases, the value is ‘1’, otherwise it is ‘0’; if the main respondent suffers from chronic lung diseases, heart disease, stroke and cancer and other malignant tumors, the value is ‘1’, otherwise it is ‘0’; Having hypertension, diabetes and other chronic diseases are considered to be suffering from mild chronic diseases, so the value is ‘1’, otherwise it is ‘0’.

#### 2.2.2. Medical Service Utilization

This index is divided into two parts: outpatient and hospitalization. According to the CHARLS questionnaire, there are the following questions: In the outpatient medical service utilization, ‘In the past month, did you go to a medical institution to see an outpatient clinic or receive on-site medical services (excluding physical examination)?’ ‘Have you ever been ill in the past month?’ Questions such as ‘What’s the main reason for you not to see a doctor’, ‘What medical institutions did you go to for outpatient treatment in the past month’, ‘How many times did you go to this medical institution in the past month” provide relevant data. In the part of hospitalization medical service utilization, ‘In the past year, did a doctor say that you should have been hospitalized’, ‘What are the main reasons why you did not go to hospital’, ‘Have you ever been hospitalized in the past year’, ‘How many hospitalizations have you received in the past year’ and other questions are asked to understand the inpatient medical service utilization of respondents.

#### 2.2.3. Medical Expenditure

Similar to the second part, the medical expenditure of the sample is divided into outpatient and hospitalization which is measured by asking ‘What was the total cost of visiting this medical institution in the past month? How much did you pay yourself’, and ‘What is the total cost of hospitalization in the past year (excluding escort, family transportation and accommodation)?’ ‘How much of it was self-paid?’

#### 2.2.4. NRCMS

Firstly, according to the data of the two periods of CHARLS, the proportion of farmers participating in NRCMS is over 91%, while about 9% do not. Therefore, the following part investigates the differences of health status, utilization of medical services and expenditure between the participating elderly and the non-participating elderly by distinguishing whether the farmers have participated in NRCMS. Secondly, combined with CHARLS, this study also investigates how NRCMS policy affects the medical service utilization and expenditure of the elderly, especially the chronic disease patients, from the dimensions of supplementary medical insurance (such as major illness medical treatment) and reimbursement methods.

#### 2.2.5. Other Variables

Other control variables also are controlled, such as travel distance, regional dummy variables, per capita family income, gender, age, education level, marital status, whether to live with children, family size (excluding the main respondents and their spouses) and other socio-economic demographic characteristics. What’s notice, it refers to the distance between the participating farmers’ medical treatment places and their homes, which not only reflects their medical treatment cost, but also the accessibility of medical service resources, the level of medical institutions and the service quality.

## 3. Empirical Results Analysis

### 3.1. Basic Sample Description and Statistics

Table 1 gives the descriptive statistics, the mean of the main variables, and Table 2 reports the situation of the rural elderly people with chronic diseases who should have seek medical treatment from both outpatient and hospitalization aspects. Table 3 reports the distribution of accessible outpatient and hospitalization care of different area in rural China.

From the perspectives of chronic disease prevalence, 84.7% of the rural elderly people suffer from chronic diseases. In particular, almost 90% of treated patients (including outpatient and hospitalization, the same below) have more than one chronic disease. More than 20% of the patients have severe chronic diseases such as chronic lung disease, heart disease, stroke and cancer, which is about 3~4% higher than that of the total sample. In terms of the mild chronic disease’s prevalence, the prevalence of all samples is about 79% while that of sick or treated samples nearly reaches 90%. These reflect the rural elderly are facing serious health threats from chronic diseases and that one of the focuses and difficulties in China’s medical and health service system reform under the accelerated population aging background is to let the elderly age healthily with proper medical care.

With regard to medical service utilization and expenditure, only 31.8% of rural elderly chronic disease patients have medical treatment behavior within a given period of time and obtain medical service utilization. According to the outpatient sample, the average number of out-patient visits per capita of rural elderly chronic disease patients is about 4 times in the past month, the total outpatient expenses is 300 yuan, and the actual outpatient expenses reimbursement ratio is 12.5%. Regarding the hospitalization sample, the average hospitalization time of rural elderly chronic disease patients is 1.6 times in the past year, the total hospitalization expense is more than 7200 yuan, but the actual reimbursement ratio is only 37.9%. This implies that most medical expenses still need to be covered by themselves and their families, resulting in a great financial burden. As Table 2 displays, economic difficulties (‘poor’) have become a critical reason why the rural elderly, who should seek medical treatment, have not seen a doctor.

As for the participation in the medical insurance system, 90% of the respondents participate in NRCMS, but only 3–4% of them participate in the supplementary medical insurance, e.g., the major illness medical insurance. About one third of the participants say that the local NRCMS has adopted immediate reimbursement rather than self-payment before reimbursement. Additionally, Table 1 also lists the basic information of age, gender, marital status, per capita family income, regional characteristics and other variables, which will not be repeated here.

### 3.2. Influence of NRCMS on Medical Service Utilization of Rural Elderly Chronic Disease Patients

When investigating the influence of NRCMS on the medical service utilization of rural elderly chronic disease patients, this paper focuses on whether they get treated and that whether they get hospitalized. The estimated results are given in Table 3 and Table 4, respectively.

#### 3.2.1. Medical Treatment Behavior

Table 4 suggests that from the perspective of chronic diseases, the chronic disease number of rural elderly people has significantly increased the probability of seeking medical treatment, which is statistically significant at the level of 1%. The results of marginal effect calculation show that when other variables remain unchanged, the probability of seeking medical treatment will rise 3.2% for every increased number of chronic diseases among the rural elderly. Although having mild chronic diseases such as hypertension and diabetes decreases the probability of seeking medical treatment, having serious chronic significantly increases the probability of medical treatment.

The impact of NRCMS on the decision-making of the rural elderly chronic disease patients is not consistent: on one hand, compared with the non-participators in NRCMS, the NRCMS insured people significantly increase the probability to seek to medical treatment. On the other hand, whether to participate in supplemental insurance to NRCMS and reimbursement method (i.e., whether to reimburse immediately) have no significant effect. It is worthy to further investigate the influence of NRCMS on the medical treatment seeking behavior of elderly patients.

In terms of socioeconomic and demographic variables, whether living with children, the travel distance to the medical facility and the residential areas significantly affect the probability of seeking medical treatment of rural elderly people with chronic diseases. The empirical result shows that the marginal effect calculation results indicate that the probability for rural patients living with their children significantly declines by 16.3% compared with those who do not. The empirical result also shows that the travel distance to the medical facility plays a significant negative role on the medical treatment behavior for elderly patients with chronic diseases. Compared with the rural elderly people with chronic diseases in the central China, the probability for patients in eastern region to seek medical treatment decreases by 10.7%. In addition, variables such as gender, age, education level, marital status, house income status and family sizes show significant effect.

#### 3.2.2. Hospitalization Behavior

Table 5 reports the regression results of the hospitalization decision-making model of rural elderly people with chronic diseases. The influences of the number of chronic diseases and the severity of the disease are not consistent with each other. The number of chronic diseases significantly increases the hospitalization tendency of rural elderly patients at the statistical level of 5%. Controlling for the other variables, the marginal effect result suggests that the probability of hospitalization significantly increase by 2.8% if they increase a kind of chronic disease. Having mild chronic diseases reduces the hospitalization probability of the rural elderly population while having severe chronic disease increases the hospitalization tendency. However, these results are not statistically significant.

From the perspective of NRCMS, the key variable of this study, whether to participate in NRCMS and whether to participate in the NRCMS’ supplemental insurance has no significant effect on the hospitalization decision-making of rural elderly people with chronic diseases. However, the immediate reimbursement significantly reduces the hospitalization probability at the statistical level of 10%. The marginal effect result implies that if other variables are controlled, immediate reimbursement could significantly reduce the hospitalization probability by 7.2%. The result indicates that immediate reimbursement improves the convenience, patients only need to pay their own expenses, which helps to increase the chances to receive NRCMS reimbursement, thus significantly increasing their willingness for hospitalization.

The travel distance from family to medical institution significantly increases the hospitalization probability of rural elderly patients with chronic diseases at the statistical level of 1%. The marginal effect result suggests that the hospitalization probability declines by about 0.2% for each additional kilometer of traffic distance when other variables are controlled. Travel distance reflects the technical level and service quality of medical institutions to some degree. On one hand, the farther the travel distance, the higher the transportation expense, the heavier financial burden of medical treatment on patients’ family; on the other hand, chronic diseases are featured with long incubation period and long illness period, making them difficult to cure. Therefore, living far away from the hospital obviously reduces their willingness to be hospitalized.

As for other variables, regional dummy variables and year variables have significant negative effects on hospitalization decision-making of rural elderly chronic disease patients. Other variables such as gender, age and education level are not statistically significant.

### 3.3. Impact of NRCMS on Medical Expenditure of the Rural Old People with Chronic Diseases

Table 6 gives the regression results of outpatient and hospitalization medical expenditure for rural elderly patients with chronic diseases.

#### 3.3.1. Outpatient Expenditure

Outpatient expenses is one of the focuses of this paper. Whether to participate in NRCMS, whether to participate in NRCMS supplementary medical insurance, reimbursement methods and outpatient reimbursement ratio have different effects on outpatient expenditure of rural elderly patients with chronic diseases. (1) The two variables of whether to participate in NRCMS and whether to participate in supplementary medical insurance have an impact on outpatient expenditure, but they are not significant. (2) Immediate reimbursement reduces the outpatient expenses of rural elderly patients with chronic diseases, and has statistical significance at the level of 1%. The marginal effect calculation suggests that, when other conditions are controlled, the immediate reimbursement significantly reduces the annual outpatient expenses of patients by nearly 190 yuan compared with the reimbursement method of self-payment before reimbursement. Because immediate reimbursement allows patients to pay only for their own medical expenses, which helps to increase their access to outpatient reimbursement, thereby reducing outpatient expenses. (3) The actual outpatient reimbursement ratio has a significant positive effect on outpatient expenses of rural elderly patients with chronic diseases at the statistical level of 10%. Controlled the other variables, the marginal effect results show the annual outpatient expenses could increase by about 940 yuan while the actual outpatient reimbursement ratio increase 1%. On one hand, the higher the reimbursement rate of NRCMS general outpatient service, the lower the sensitivity of the rural elderly patients to the medical service price change; on the other hand, they have a strong willingness to avoid health risks. Besides, most of them have only received private schools or even no formal education, they are unable to understand the medical information related to chronic diseases but mainly rely on medical service providers to obtain disease-related information and easily get induced, thereby increasing outpatient expenditure.

In terms of other variables, travel distance increases the outpatient expenses of rural elderly patients with chronic diseases. Compared with male, the outpatient expenses of female patients increase significantly, because women are more vulnerable to gynecological chronic diseases and are more willing to go to the clinic, thus increasing outpatient costs. With regard to regional variables, compared with the rural elderly patients in central China, the outpatient expenses of those in eastern China increase significantly. The number of chronic diseases, mild chronic diseases, severe chronic diseases and other socioeconomic and demographic variables are not statistically significant.

#### 3.3.2. Hospitalization Expenditure

In terms of hospitalization expenditure, the number of chronic diseases would increase the hospitalization expenditure of rural elderly patients with chronic diseases, but whether the chronic diseases are mild or severe is not statistically significant.

From the perspective of NRCMS, whether to participate in NRCMS reduces the hospitalization expenditure of elderly patients with chronic disease, whether to participate in NRCMS supplementary medical insurance, immediate reimbursement and the actual hospitalization reimbursement ratio increase the hospitalization expenditure, however, these conclusions are not statistically significant. It means that NRCMS hospitalization compensation policy has no obvious effect on reducing hospitalization expenses. The reason may be that chronic diseases, characterized by long duration and frequent occurrence, need regular treatment. NRCMS hospitalization compensation policy is mainly to pay for serious illness and hospitalization and provide less compensation for chronic disease expenses.

Travel distance has a positive effect on hospitalization expenditure of rural elderly patients with chronic disease, which is statistically significant at the level of 1%. Due to the underdeveloped public health in rural areas and the insufficient chronic disease management. In the case of imperfect three-level medical and health care system and concentrated medical resources in urban or economically developed areas, rural patients tend to seek higher-level treatment and often have to choose large general hospitals, which increases the expenditure of medical treatment and hospitalization.

## 4. Discussion

This study tries to investigate the impact of NRCMS on the medical service utilization for elderly people with chronic disease in rural China. The empirical results have shown that the rural elderly people still remain poor health status and have difficulty obtaining access to care utilization with chronic disease. We also find when someone has no money, they prevent seeking to medical treatment and hospitalization. Although China’s government established the NRCMS and the NRCMS’ supplemental insurance, liking critical illness insurance, for rural residents, the patients pay for medical treatment after getting sick. Moreover, the insured patients apply for medical assistance is for a specific group of people and specific diseases. If the family is poor, it is usually through borrowing money from their relatives and friends, and only a small number of people can apply for medical assistance.

Some policy implications could be taken. The first is the health status of the rural elder, especially the prevention and control of chronic diseases. At present, in China, especially in rural areas, the elder is facing a serious health threat-chronic disease, resulting in a heavy financial burden. Facing the rapid rural population aging, how to effectively deal with chronic diseases and their social and economic impact becomes the focus of relevant policies in the long run. The second focus is how to improve NRCMS and deepen the reform of medical and health service system so that the rural elderly chronic disease patients can receive appropriate treatment. Although NRCMS improves the hospitalization services to some extent, they still have high hospitalization expenditure due to small compensation scope, low compensation level and induced demand of medical service providers. NRCMS cannot effectively meet the needs of rural elderly chronic disease patients. It is necessary to deepen the reform of medical security system represented by NRCMS, improve the public health service system, and strengthen its operation effect to handle the challenges of chronic diseases.

Furthermore, by 2050, the world’s population aged 60 years and older is expected to total 2 billion, and 80% of all older people will live in low- and middle-income countries. Moreover, there will be almost 434 million people in this age group worldwide by 2050. There is, however, little evidence to suggest that older people today are experiencing their later years in better health than their parents. Common conditions in older age include chronic obstructive pulmonary disease, diabetes, depression, and dementia so on. Furthermore, as people age, they are more likely to experience several conditions at the same time. Chronically ill older adults must integrate self-care behaviors into their daily routine to promote health and reduce urgent health care utilization [33]. Policymakers should pay more attention on the chronic elder in rural area. Some studies insisted that in additional to need and financial resources, psychosocial variables may be particularly influential for underutilized services [34]. They should not only better meet the health demand of the rural elderly, but also deepen the current health system reform and adopt a combination of medical care and old-age care. They should implement the strategy and specific actions of ‘integrating health into all policies’ in order to provide health care for the elder, extend the life span and turn the aging population into valuable wealth [35].

Health systems need to be better organized around older people’s needs and preferences, designed to enhance older people’s intrinsic capacity, and integrated across settings and care providers. However, to our knowledge, medical services for chronic non-communicable diseases and their treatment have not been covered by medical insurance. Luckily, in 2016, China’s government has implemented the pilot work to merge the NRCMS and the Urban Residents’ Basic Medical Insurance System as a united basic health insurance. China’s government further continues to advance the reform of the medical and health system and medical insurance, focusing on medical insurance payment methods, endemic diseases, chronic diseases, and special populations.

Finally, it should be pointed out that due to the limited data availability, this paper does not include the factors of demand induced by medical service providers into the analysis framework, nor does it distinguish the impact of medical resource adequacy on medical service utilization and the financial burden of chronic elder in rural China. These questions further need to be answered by offering more theoretical and empirical evidence. For example, a recent study has played the eyes on internet use and mental health resulting from the chronic diseases among the elderly in Shanghai, China [36].

## 5. Conclusions

By adopting the data of CHARLS in 2011 and 2013, this research focuses on the influence of NRCMS on the medical service utilization and expenditure of chronic elder in rural China. More than 85% of the rural elder suffer from at least one chronic disease, and one fifth of the elder suffer from severe chronic diseases such as heart disease, stroke, chronic lung disease and cancer. In additional, NRCMS does not significantly change the hospitalization probability of elderly chronic disease patients in the decision-making. NRCMS immediate reimbursement significantly increases the hospitalization probability, but whether to participate in NRCMS and whether to participate in supplementary medical insurance do not. It is worthy that immediate reimbursement significantly reduces the outpatient expenses of rural elderly chronic disease patients, but the actual outpatient reimbursement ratio increases their outpatient expenditure significantly. In addition, since the NRCMS policy is mainly designed to pay for major illness and hospitalization at the beginning with less compensation for chronic diseases, the effect of NRCMS on hospitalization expenses of rural elderly people with chronic diseases is not significantly.

China’s rural older with chronic diseases place heavy economic burden on the households associated with frequent outpatient visits and use of multiple prescription medicines. Our results showed that NRCMS played an important role on financial protection for the rural elderly and their household with chronic disease. However, the NRCMS policies in rural China should be strengthened in the future.

## Figures and Tables

**Table 1:** Basic descriptive statistics of main variables, mean (SD).

Variable	Total Sample	Sick Sample	Treated Sample	
			Outpatient	Hospitalization
**Chronic Disease:**				
Having a Chronic Disease (1 = Yes)	0.847 (0.360)	1.000 (0.000)	0.924 (0.265)	0.944 (0.231)
Number of Chronic Diseases	1.794 (1.391)	2.116 (1.265)	2.147 (1.429)	2.379 (1.480)
Mild Chronic Disease (1 = Yes)	0.794 (0.404)	0.937 (0.244)	0.875 (0.331)	0.880 (0.325)
Serious Chronic Disease (1 = Yes)	0.171 (0.376)	0.201 (0.401)	0.204 (0.403)	0.209 (0.407)
**Medical Service Utilization and Expenditure:**				
Having Medical Treatment (1 = Yes)	0.318 (0.466)	0.347 (0.476)	--	--
Times of Outpatient Visit	--	--	3.748 (5.069)	--
Outpatient Expense (10,000 yuan)	--	--	0.030 (0.110)	--
Actual Outpatient Expenses Reimbursement Ratio (%)	--	--	0.125 (0.172)	--
Times of Hospitalization	--	--	--	1.600 (1.683)
Hospitalization Expense (10,000 yuan)	--	--	--	0.726 (1.254)
Actual Hospitalization Expenses Reimbursement Ratio (%)	--	--	--	0.379 (0.282)
Travel Distance (km)	--	--	9.178 (21.192)	24.155 (33.499)
**NRCMS:**				
Participating in NRCMS (1 = Yes)	0.898 (0.303)	0.899 (0.301)	0.909 (0.288)	0.897 (0.304)
Participating in Supplemental Insurance to NRCMS (1 = Yes)	--	0.041 (0.198)	0.037 (0.189)	0.040 (0.196)
Immediate Reimbursement (1 = Yes)	--	0.296 (0.456)	0.309 (0.462)	0.236 (0.425)
**Socio-economic Demographic Characteristics:**				
Age (Years Old)	69.002 (7.087)	68.743 (6.880)	68.699 (6.809)	68.993 (6.655)
Male (1 = Yes)	0.528 (0.499)	0.525 (0.499)	0.505 (0.500)	0.545 (0.499)
Primary School Education or Above (1 = Yes)	0.365 (0.481)	0.369 (0.483)	0.361 (0.480)	0.402 (0.491)
With Spouse (1 = Yes)	0.637 (0.481)	0.673 (0.469)	0.654 (0.476)	0.688 (0.464)
Family Size (People)	2.470 (2.170)	2.559 (2.166)	2.628 (2.390)	2.618 (2.122)
Living with Children (1 = Yes)	0.269 (0.444)	0.232 (0.422)	0.228 (0.420)	0.199 (0.400)
Per Capita Income (10,000 yuan)	0.306 (0.589)	0.284 (0.546)	0.319 (0.636)	0.287 (0.423)
Central (1 = Yes)	0.363 (0.481)	0.359 (0.480)	0.385 (0.487)	0.319 (0.467)
Eastern (1 = Yes)	0.310 (0.462)	0.300 (0.458)	0.247 (0.431)	0.259 (0.439)
Western (1 = Yes)	0.327 (0.469)	0.341 (0.476)	0.368 (0.483)	0.422 (0.495)
Year (1 = Year 2013)	0.416 (0.493)	0.491 (0.500)	0.459 (0.499)	0.556 (0.498)
Instrumental Variable				
Provincial NRCMS Participation Rate	0.686 (0.140)	0.687 (0.142)	0.701 (0.147)	0.689 (0.138)
Observed Sample	2418	2050	592	301

Notes: (1) Values in brackets are standard variance and “--” means non-existence. (2) Among the sick samples, outpatient samples and hospitalized samples, the values of the two variables of New Rural Cooperative Medical System (NRCMS) supplementary insurance and immediate reimbursement refer to the relevant values of NRCMS participants. (3) It should be noted that 124 people have both outpatient and hospitalization behaviors in the survey year.

**Table 2:** Situations of the rural elderly who should have been treated.

Outpatient			Hospitalization		
Reason	Numbers	Percent	Reason	Numbers	Percent
Already Under Treatment	30	13.39	Poor	85	61.59
Illness is Not Serious	92	41.07	Not Willing	24	17.39
Poor	48	21.43	Unlikely to cure and Hospital Quality Poor	5	3.62
No Time	1	0.45	Unlikely to cure and Problem too Serious	4	2.90
Inconvenient Traffic	12	5.36	Others	20	14.49
Poor Service	1	0.45			
No Available Treatment	7	3.13			
Others	33	14.73			

**Table 3:** Distribution of accessible outpatient and hospitalization care.

	Outpatient		Hospitalization	
	Mean	Obs	Mean	Obs
Central China	0.260 (0.439)	878	0.109 (0.312)	877
East China	0.196 (0.391)	745	0.104 (0.306)	747
West China	0.276 (0.447)	790	0.161 (0.367)	791

**Table 4:** Results of medical treatment behavior for elderly people with chronic diseases.

	First-Stage	Second-Stage	
	Participating in NRCMS	Having Medical Treatment	
	Coefficient	Coefficient	Marginal Effect
Provincial NRCMS Participation Rate	0.100 * (0.045)	--	--
Participating in NRCMS	--	8.947 *** (2.518)	3.074 ***
Supplemental Insurance to NRCMS	--	0.036 (0.144)	0.012
Immediate Reimbursement	--	−0.101 (0.068)	−0.035
Number of Chronic Diseases	0.001 (0.006)	0.094 *** (0.021)	0.032 ***
Mild Chronic Disease	0.001 (0.098)	−0.021 (0.140)	−0.007
Serious Chronic Disease	−0.034 (0.023)	0.373 *** (0.121)	0.128 ***
Travel Distance	--	−0.031 *** (0.003)	−0.010 ***
Male	−0.039 *** (0.015)	0.296 *** (0.106)	0.102 ***
Age	−0.005 *** (0.001)	0.047 *** (0.012)	0.016 ***
Primary School Education or Above	−0.012 (0.016)	0.179 ** (0.072)	0.061 **
With Spouse	0.012 (0.019)	−0.047 (0.082)	−0.016
Family Size	−0.005 (0.004)	0.064 *** (0.020)	0.022 ***
Living with Children	0.023 (0.022)	−0.473 *** (0.098)	−0.163 ***
Per Capita Income	−0.073 *** (0.020)	0.579 *** (0.144)	0.199 ***
Eastern	0.012 (0.021)	−0.346 *** (0.087)	−0.107 ***
Western	0.019 (0.017)	0.076 (0.084)	0.029
2013	−0.024 (0.023)	0.154 (0.120)	0.053
Constant	0.213 ** (0.106)	−12.622 *** (3.155)	--
Residual	--	−8.881 *** (2.513)	−3.052 ***
Log pseudolikelihood	−417.519	−1264.260	
Scale parameter	0.089	0.203	
AIC	0.422	1.252	
BIC	−15,337.710	−15,075.530	
Observed Sample	2050	2050	

Notes: (1) The values in brackets are standard errors, calculated by 500 times bootstrapping. ‘--’ means non-existence. (2) ***, ** and * represent statistical significance at the level of 1%, 5% and 10%, respectively.

**Table 5:** Results of hospitalization behavior of elderly people with chronic diseases.

	First-Stage	Second-Stage	
	Participating in NRCMS	Hospitalization	
	Coefficient	Coefficient	Marginal Effect
Provincial NRCMS Participation Rate	0.100 * (0.045)	--	--
Participating in NRCMS	--	−2.835 (3.369)	−1.138
Supplemental Insurance to NRCMS	--	−0.070 (0.195)	−0.028
Immediate Reimbursement	--	−0.179 * (0.109)	−0.072 *
Number of Chronic Diseases	0.001 (0.006)	0.071 ** (0.032)	0.028 **
Mild Chronic Disease	0.001 (0.098)	−0.269 (0.174)	−0.108
Serious Chronic Disease	−0.034 (0.023)	0.142 (0.225)	0.057
Travel Distance	--	0.004 *** (0.001)	0.002 ***
Male	−0.039 *** (0.015)	−0.140 (0.154)	−0.056
Age	−0.005 *** (0.001)	−0.005 (0.016)	−0.002
Primary School Education or Above	−0.012 (0.016)	0.105 (0.107)	0.042
With Spouse	0.012 (0.019)	−0.001 (0.125)	−0.0004
Family Size	−0.005 (0.004)	−0.036 (0.029)	−0.014
Living with Children	0.023 (0.022)	0.270 (0.187)	0.108
Per Capita Income	−0.073 *** (0.020)	−0.269 (0.219)	−0.108
Eastern	0.012 (0.021)	0.248 * (0.135)	0.092 *
Western	0.019 (0.017)	0.354 *** (0.125)	0.139 ***
2013	−0.024 (0.023)	0.506 ** (0.218)	0.203 **
Constant Term	0.213 ** (0.106)	1.647 (4.306)	--
Residual	--	2.963 (3.334)	1.189
Log pseudolikelihood	−417.519	−470.575	
Scale parameter	0.089	0.226	
AIC	0.422	1.375	
BIC	−15,337.710	−4395.337	
Observed Sample	2050	712	

Notes: (1) The values in brackets are standard errors, calculated by 500 times bootstrapping. ‘--’ means non-existence. (2) ***, ** and * represent statistical significance at the level of 1%, 5% and 10%, respectively.

**Table 6:** Results of medical expenditure of rural elderly people with chronic diseases.

Variable	Outpatient Expenditure	Hospitalization Expenditure
Number of Chronic Diseases	0.001 (0.003)	0.026 (0.065)
Mild Chronic Disease	0.007 (0.023)	−0.165 (0.291)
Serious Chronic Disease	−0.009 (0.015)	−0.252 (0.262)
Participating in NRCMS	−0.023 (0.022)	−0.202 (0.235)
Supplementary Insurance to NRCMS	0.020 (0.015)	0.119 (0.388)
Immediate Reimbursement	−0.019 *** (0.008)	0.056 (0.197)
Actual Reimbursement Ratio	0.094 *** (0.031)	0.016 (0.416)
Travel Distance	0.0004 (0.0004)	0.009 ** (0.004)
Male	−0.043 ** (0.020)	0.400 * (0.248)
Age	−0.003 (0.001)	0.003 (0.024)
Primary School Education or Above	0.006 (0.016)	−0.133 (0.239)
With Spouse	0.020 ** (0.010)	0.040 (0.160)
Family Size	−0.001 (0.003)	0.0003 (0.046)
Living with Children	0.002 (0.014)	−0.103 (0.248)
Per Capita Income	−0.035 ** (0.018)	0.040 (0.415)
Eastern	0.019 * (0.012)	0.464 * (0.267)
Western	0.020 (0.014)	−0.072 (0.189)
2013	−0.005 (0.016)	0.207 (0.240)
Constant Term	0.672 ** (0.348)	−1.526 (6.904)
Residual	−0.469 * (0.263)	1.964 (5.766)
Log pseudolikelihood	−678.222	−386.402
Scale parameter	0.005	0.957
AIC	2.410	2.862
BIC	−3326.066	−1238.62
Observed Sample	551	285

Notes: (1) The values in brackets are standard errors, calculated by 500 times bootstrapping. (2) ***, ** and * represent statistical significance at the level of 1%, 5% and 10%, respectively.
